# LeaData a novel reference data of leather images for automatic species identification

**DOI:** 10.1038/s41598-025-88040-1

**Published:** 2025-02-06

**Authors:** Anjli Varghese, Malathy Jawahar, A. Amalin Prince

**Affiliations:** 1 Department of Electrical and Electronics Engineering, BITS Pilani, K K Birla Goa Campus, Goa, 403726 India; 2 Central Leather Research Institute, Chennai, 600020 India

**Keywords:** Digital microscope, Grain patterns, Image analysis, LeaData, Leather images, Species identification, Engineering, Mathematics and computing

## Abstract

In the leather industry, the mammalian skins of buffalo, cow, goat, and sheep are the permissible materials for leather-making. They serve the trade of quality leather products; hence, the knowledge of animal species in leather is inevitable. The traditional identification techniques are prone to ambiguous predictions due to insufficient reference studies. Indeed, leather image analysis with big data can pave the way for automatic and objective analysis with accurate prediction. This study focuses on creating novel and unique leather image data, LeaData. The objective is to automatically determine species from grain surface analysis. Hence, it employs a simple, cheaper, handheld digital microscope for leather image acquisition. The magnifying parameter 47$\times$ captures the species-unique grain patterns distributed over the leather surface. In total, the LeaData encloses 38,172 images of four species from 137 leather samples. This big data spans leather images with theoretically ideal and practically non-ideal grain patterns. It also includes images of grain patterns varying over different body parts. Thus, the novel LeaData is an adequately larger pool of leather images with diverse behavior. The motive is to establish a smart leather species identification technique that can be easily accessible by leather specialists, customs officials, and leather product manufacturers. Hence, this paper solely creates the bigger LeaData and presents its different versions to the digital image processing and computer vision research community. This digitized source of permissible leather species helps enable digitization in leather technology for species identification. In turn, in maintaining biodiversity preservation and consumer protection.

## Introduction

Leather made from animal skin/hide is a highly durable and versatile material, and its products serve a great economic interest^1^. The export/import of good quality leather helps the country’s consistent growth in the export arena and thereby improve the country’s economy^2^. The leather quality depends on the animal species, age, gender, region of origin, portion of the skin/hide (body part), and the type of leather. However, species of origin is the primary factor that defines the quality of leather.

In the leather industry, the hides/skins of the most commonly reared domestic species, such as buffalo, cow, goat, and sheep, are the permissible materials for leather-making. However, the skins of several exotic animals, namely crocodile, ostrich, lizard, snake, etc., are used in the manufacture of luxury leather products^3^. Also, low-grade animal skins mixed with fake materials are used in the production of faux leather^4^. These practices are non-permissible as they can cause a significant loss in biodiversity and leather export share. Reliable information regarding leather species can help prevent the trade of leather from non-permissible animal skin and protect the consumers from fraudulence, counterfeiting, mislabeling, etc.^5^.

**Fig. 1:**
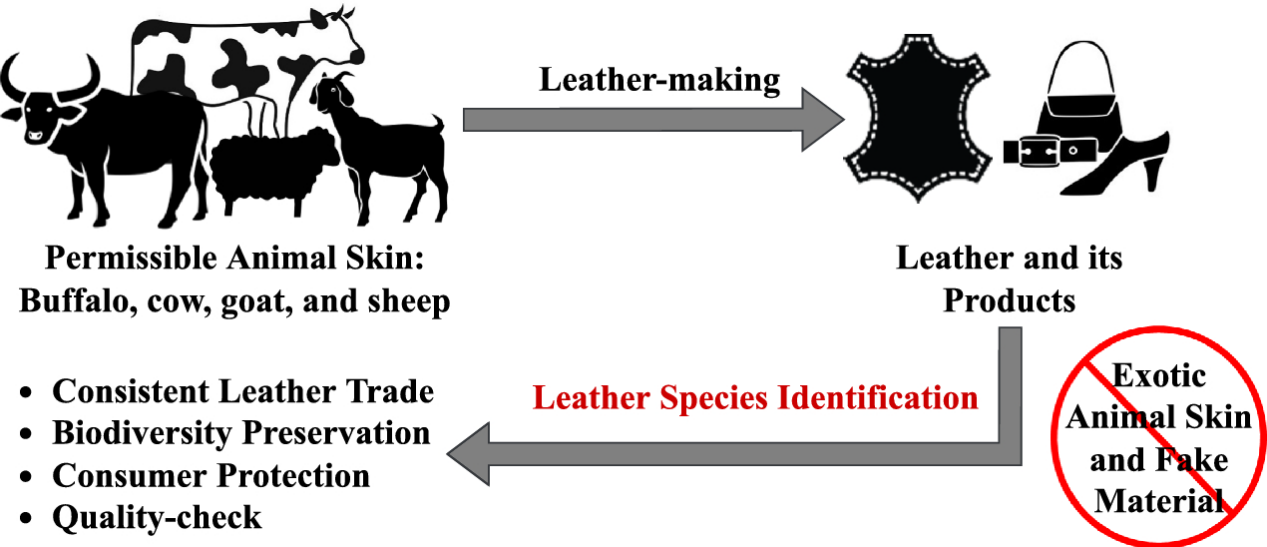
Background for leather species identification.

Moreover, among different permissible species, the raw material is chosen based on the final leather product requirement. Because the physical properties of leather vary among each species^6^. The leather from buffalo hide and goatskin is firm and rigid, and cowhide and sheepskin are soft and supple. Also, globally, cowhides and sheepskins are considered the universal raw materials for leather-making. At the same time, the buffalo hides and goatskins are confined to a few countries^7^. That is, the source of a leather product is consumer and product-specific, and the act of substituting the raw materials with incorrect and fake materials is against the Consumer Protection Act^5,8^. Therefore, it is inevitable for the supplier to specify the leather species before the product is manufactured and traded. In other words, knowledge of animal species is also a necessary factor in making leather products fulfilling their requirements. On the whole, in the leather industry, species identification is a crucial step in maintaining consistent global leather trade, consumer protection, biodiversity preservation, and quality-check. It holds a crucial role in certifying the authenticity, permissibility, value, and quality of the leather and its products. Figure 1 schematically overviews the background for leather species identification.

Different leather species identification techniques are in practice. Among these, macroscopic examination of the physical properties of a leather sample is one of the traditional methods^7^. This method can often face erroneous decisions due to leather expert-dependent judgment and knowledge insufficiency. In contrast, the biochemical approaches analyzed the DNA-based genetic information and collagen-based protein structures^8,9^ for effective species prediction. However, the DNA degraded during the leather-making process can cause misinterpretations, and the collagen analysis is economically inefficient due to the highly expensive and bulky setup. Meanwhile, microscopic examination of the grain patterns on the leather surface is a comparatively less complex method^10^. Yet, the knowledge of structural properties of grain patterns developed over generations is insufficient for firm/objective decisions. On the other hand, analytically analyzing the images of leather with grain patterns can provide objective predictions. However, highly expensive image sensing devices and tedious data curation process are the major shortcomings. Therefore, practically, the conventional leather species identification techniques are theoretical, subjective, and supervised. They encounter biased decisions due to inadequate reference studies. Hence, this research aims to develop a complete reference study of grain patterns of finished leather samples for automatic identification of species of origin.

In today’s digital world, learning, analyzing, and taking actions from images by means of a computer can emulate human intelligence with objective decisions. The present work proposes to analyze leather images to facilitate a human-machine interactive platform in the leather industry for automatic species identification. Henceforth, it establishes a novel and unique digital microscopic leather image data (LeaData). A cost-effective, compact, and simple digital microscope with 47$\times$ magnification and 1024 $\times$ 1280 image resolution is used for leather image acquisition. It acquires grain surface images of four permissible species: buffalo, cow, goat, and sheep. The grain/hair pore and its pattern are distinct to each species. The pores are the smallest in the sheep, followed by the goat, cow, and buffalo receptively. Meanwhile, the pattern of pore distribution is wavery in the sheep, clustered in the goat, densely packed in the cow, and scattered in the buffalo images. But, practically, the grain patterns possess vivid characteristics that may vary between different leather samples. These variations are due to the animal species raised in different regions of locations, breed, age, gender, etc. Hence, the LeaData encloses the grain surface images of four species from 137 different leather samples. Moreover, it includes images of grain patterns with theoretically ideal and practically non-ideal behavior. Yet, it is challenging to determine species from different parts of the animal skin. Because compact to stretchy grain patterns are noticeable from one body part to another. Henceforth, the LeaData spans grain surface images of four species from six body parts: backbone, belly, butt, neck, shank, and shoulder. Therefore, unlike the traditional data, the novel LeaData is a bigger data comprising 38,172 leather images. It is an adequate reference data for permissible leather species prediction.

Furthermore, this big data, the LeaData with vivid behavior, is sub-grouped into three different versions: LeaData-V1, LeaData-V2, and LeaData-V3. Each version includes leather images with ideal, non-ideal, and body part-specific behavior, respectively. LeaData-V1 is created as the smallest data with 1,200 images suitable for basic digital image processing (DIP) and machine learning (ML) applications. In contrast, LeaData-V2 is a comparatively bigger data with 7,600 images developed for deep learning (DL) based applications. The LeaData-V1 and LeaData-V2 enclose images from one part of the body, i.e., the butt. Meanwhile, the LeaData-V3 is the largest data with 10K images acquired from different body parts. It is well-suited for complex DL applications with diverse and big data analysis. The images of LeaData-V1 and LeaData-V2 are made openly available in the Dryad data repository. Thus, this research presents novel, unique, and bigger data to the DIP and computer vision fields to establish the digitalization process in leather technology for automatic and digitized leather species identification. This digitalization can assist leather specialists, customs officials, and leather product manufacturers with quick and accurate species determination. In turn, it can pave the way for biodiversity preservation and consumer protection by ensuring the trade of leather from permissible materials.

The rest of the paper is organized into the following sections: Section 2 discusses the various traditional leather species identification methods and highlights their shortcomings. Section 3 gives a detailed description of the acquisition process of the present LeaData and the characteristic diversity in the leather images. Section 4 describes the different versions of LeaData and the recent methodologies explored and proposed using the novel LeaData. Finally, Section 5 summarizes the scope of LeaData in the leather technology, DIP, and computer vision fields.

## Related work

In the leather industry, identifying the species of origin in leather is an inevitable process to preserve biodiversity, prevent fraudulence, and maintain consistent and trustworthy trade. Earlier, the leather specialists determined species by physically observing the leather size (weight and thickness), grain patterns on the leather surface, and feeling the leather texture (roughness/softness)^7,8^. It is the traditional species identification process supervised by well-trained experts. Eventually, due to the diverse species’ characteristics, the knowledge gained by the experts became insufficient. The leather surface damage caused during the leather-making processes also led to ambiguous judgment. This theoretical, expert-dependent, traditional method with inaccurate decision demanded the other strong species identification methods^5^. Accordingly, research on leather species identification using biochemical analysis, microscopic visual analysis, and leather image analysis has emerged.

### Biochemical analysis

Generally, the information contained in DNA, enzymes, proteins, etc., are unique to each species. Determining species uniqueness in leather by analyzing the DNA and protein structure can be referred to as biochemical analysis. Over the decades, different studies on DNA and collagen-based biochemical analyses have been conducted for precise leather species identification.

Deoxyribonucleic acid (DNA) analysis is a molecular-based leather species identification process. It gives the potential information on genetic patterns specific to different species. Ancient DNA (aDNA) can reflect the long-term historical assessment of the genetic information of an animal. In a study, the ancient DNA of neolithic legging was extracted to identify the animal skin used in its manufacture^11^. A similar study analyzed aDNA to identify the animal species from the remains of leather clothes like coats and bracers, tools like quivers, etc., of the bronze age^12^. Meanwhile, another study conducted aDNA analysis to identify the leather from Asiatic black bears and Felidae fur skin to prevent the illegal trade and conserve species and biodiversity^13^. Similarly, DNA analysis was practiced to identify the species of origin in the leather heads of the ancient piano in order to restore Beethoven’s music^14^. The studies discussed revealed that the leather materials used in the earlier ages were made of cattle, sheep, and goat skins and not of wild animals’ skins. Thus, DNA analysis helped the archaeologists address the bio-geographic origin of species in ancient leather and their restoration. Moreover, in the commercial aspect, DNA analysis has gained significant attention in fraud detection and quality control in leather^9^. However, denaturation, the chemical treatment of leather at high temperatures, and coloring processes can cause DNA degradation^10^. Meanwhile, polymerase chain reaction (PCR) helped to retrieve the degraded DNA from colored leather samples at the cost of a tedious, complex, resource-intensive, and expert-dependent approach^3^. Yet, the lack of DNA strands (database) caused the approach to be more problematic and controversial.

On the other hand, unlike DNA, collagen is less affected by the physical and chemical treatment of leather. Collagen is the major component of protein in leather, and the protein structure is unique to each species^6^. Henceforth, different collagen analysis-based species identification methods have been developed to address the drawbacks of DNA analysis. Liquid chromatography-mass spectrometry (LC-MS) is one such method that relies on analyzing the collagen-derived peptides from the dermal layer of animal skin. A study employed the LC-MS method to differentiate among the six mammalian species: cow, deer, goat, horse, pig, and sheep^15^. Similarly, it was used in another study to prevent the mislabeling of expensive crocodilian leather samples with reasonable mammalian samples^16^. However, the limited reference study, tedious approach, and extremely expensive and huge peptide-analyzing instruments are the major drawbacks. Meanwhile, Zooarchaeology by mass spectrometry (ZooMS) is a comparatively rapid and simple collagen analysis method^17,18^. It was used to identify species in the archaeological leather remains of the medieval and Scythian ages. Unlike aDNA, ZooMS required a very small portion of the leather sample for species identification. However, the cost-ineffective and laboratory-specific huge devices and human-intervened decisions are the major limitations.

**Table 1:** Summary of conventional biochemical analyses for leather species identification.

Method	Data Analyzed	Drawbacks	Data Adequacy	Analysis
DNA analysis	DNA strands of ancient leather samples	DNA degradation due to chemical treatment, complex and tedious process	Inadequate	Subjective, expert-dependent
LC-MS and ZooMS-based collagen analysis	Collagen-derived peptides of ancient leather samples	Resource-intensive and extremely expensive and bulky setup	Inadequate	Subjective, expert-dependent
ELISA-based enzyme analysis	Enzymes of ancient leather samples	Complex and tedious procedure	Inadequate	Subjective,expert-dependent

In the scientific field, enzyme-linked immunosorbent assay (ELISA) is the widely and popularly used powerful and low-cost species diagnostic tool among different immunological techniques. A study focused on combining the ELISA approach with certain analytical methods like energy dispersive spectrometer (EDS) and attenuated total reflection Fourier transform infrared (ATR-FTIR) spectroscopy in the analysis of ancient leather^19^. The combined analysis led to a highly efficient anti-collagen antibody analysis. However, the collective method is expert-dependent, slow, and tedious. Another study conducted thermogravimetric analysis and analytical pyrolysis to decompose the materials in leather for species identification^5^. The former is a thermal technique, and the latter is a mass spectrometer-based method. The combined approach efficiently identified the raw material and the tanning process employed during the leather manufacture. Yet, the process is complex and unsuitable for finished leather samples. Thus, the biochemical analyses discussed are tedious, complex, resource-intensive, and expert-dependent. Moreover, they are mainly focused on inspecting species in the archaeological leather, and it is practically challenging to determine species from modern leather samples. Table 1 summarizes the shortcomings of the leather species identification using biochemical analyses.

### Microscopic visual analysis

In leather, the hair pores on the grain surface vary between the animal species^7^. It possesses the largest, medium-sized, medium and small-sized, and smallest pores in the buffalo, cow, goat, and sheep leather samples, respectively. Compared to biochemical analyses, examining grain surfaces is an easy means of species identification. However, the macroscopic examination of grain surfaces is inconvenient for predicting species from finished leather samples. Because, in the finished leather, due to various finishing processes, the grain patterns are barely visible to the naked eye. In such a case, the microscopic visual examination is an alternate solution to view the morphological characteristics of the grain surface easily.

Different microscopes, such as electron, stereo, optical, fluorescence, etc., are available for microscopic examination. A leather sample viewed under a stereo microscope will show uneven grain patterns, resulting in ambiguous decisions^17^. On the other hand, a scanning electron microscope (SEM) is one of the most powerful imaging devices with high magnification. In a recent study, SEM-based microscopic analysis was conducted to determine the morphological characteristics of leather from non-halal animal species^10^. It combined SEM-based visual analysis with Fourier transform infrared (FTIR) spectroscopy-based analytical method. The method utilized FTIR spectroscopy to analyze the spectral information of leather samples. Although the work framed a rapid and accurate authentication of leather and its products, it was highly expensive. The accessibility of a cost-effective grain feature diagnostic appliance is a potential problem for leather specialists. Meanwhile, a digital camera is a reasonable diagnostic tool. In a study, reflectance transformation imaging (RTI), a digital imaging technique, was proposed as an online tool for the visual analysis of leather grain surfaces^20^. It employed an Oxford RTI illumination dome equipped with LED computer-controlled light sources and a high-resolution NIKON DSLR camera to capture multiple high-quality images. The 3D appearance of images in RTI viewer experienced natural visualization. However, the magnification of images captured using a digital camera is uncertain and, the provision of setting a desired magnification is not available. Moreover, insufficient reference data and the intervention of well-trained leather specialists for the right species prediction are the other major drawbacks.

### Leather image analysis

Leather species identification techniques based on biochemical and visual analysis are human-intervened, subjective, and theoretical. Meanwhile, applying image analysis techniques to the leather images used for visual examination can provide objective and automatic prediction. A recent study proposed the automatic identification of leather species by exploiting the application of digital image processing on the SEM leather images^7^. The species-defining pores were initially isolated and segmented using an image thresholding operation. Following this, specific mathematical algorithms quantified the morphological features, such as the pore area and diameter, pore shape, pore density, and the distance between the pores. These features were mathematically quantified to objectively validate the uniqueness of each leather species. Although the method offered objective feature analysis, it relied on experts for pore feature classification.

**Table 2:** Summary of conventional microscopic visual and leather image analyses for leather species identification.

Method	Data Analyzed	Drawbacks	Data Adequacy	Analysis
SEM-based visual analysis	Well-magnified microscopic leather images of finished leather samples	Highly expensive and laboratory-specific bulky setup	Inadequate	Objective, expert-dependent
RTI-based visual analysis	Digital leather images of tanned leather samples	Complex and tedious procedure with a bulky setup	Inadequate	Objective, expert-dependent
Leather image analysis	Digital leather images of tanned leather samples	Unsuitable for finished leather image analysis with poor/uncertain magnification	Inadequate	Objective, expert-independent

Applying different learning models can facilitate the automatic classification of leather images without human intervention. Recently, a backpropagation neural network (BPNN) based learning model was utilized to automatically distinguish between the leather and non-leather samples^21^. The study analyzed the digital camera-captured leather images of cow, elephant, pig, and non-leather samples. The analysis involved Otsu’s thresholding and k-means clustering methods to derive the statistical features of the leather images. Finally, BPNN was trained to automatically classify these features into respective classes. However, modeling BPNN is tedious, and accurate classification is dependent on a large number of varied leather images. Another study adopted a convolutional neural network (CNN) to derive the features from camera-captured leather images^22^. It aimed to differentiate between mammalian (cow, goat, and sheep) and crocodilian (crocodile and lizard) leather samples. It also emphasized the features derived from a pre-trained CNN to serve a high degree of accurate classification. A linear support vector machine (SVM) based learning model was adopted for the automatic classification of CNN-derived leather image features. Yet, the study focused on the quality check and restricted to tanned leather images. Thus, the existing methodologies^21,22^ highlighted the significance of image analysis for efficient and objective decision-making. However, they are confined to tanned leather image analysis. In other words, the image analyses discussed are unsuitable for predicting species from finished leather images. Also, inadequate reference data and the use of digital cameras with poor magnification are the other significant drawbacks. Table 2 summarizes the limitations of the recent leather species identification techniques using microscopic visual and image analyses.

## LeaData creation

The mammalian skins by origin are covered by numerous hair^6^. During the leather-making procedure, the hairs are removed, and the empty hair follicles make the topmost grain surface layer of the leather sample. They form a porous structure, and their arrangement is distinct and characterizes each animal species. Leather species identification from the hair-pore examination is the least costly method^23^. However, physical observation of pore patterns from finished leather is inappropriate as the pores are not visible to the naked eye. In contrast, microscopes can view the sample details with better magnification. Analyzing microscopic images can provide objective/firm decisions and can further lead to automation. Hence, the major objective of this work is to acquire microscopic leather images for automatic/smart species identification with objective analysis.

### Leather image sensing and acquisition

In general, the property of magnified detailing of minute structures standout microscopes from other imaging devices. Conventionally, optical microscopes and SEM are widely used at the cost of huge, laboratory-specific, and expensive setups. On the other hand, a digital microscope is a handheld, compact, and portable imaging device^24^. It can capture a well-magnified image sample in a larger field of view than on an optical microscope^25^. Moreover, it is a cost-effective imaging device capable of sensing, acquiring, and displaying images in real-time^26^. The growth of image analysis using a digital microscope is extensively seen in the fields of biology, medicine, forensics, etc. The image detailing, the power of magnification, image resolution, cost-effectiveness, and compactness make the digital microscope more advantageous than the other diagnostic tools. Hence, this research proposed to use an economically efficient digital microscope for digital microscopic leather image acquisition.

**Fig. 2:**
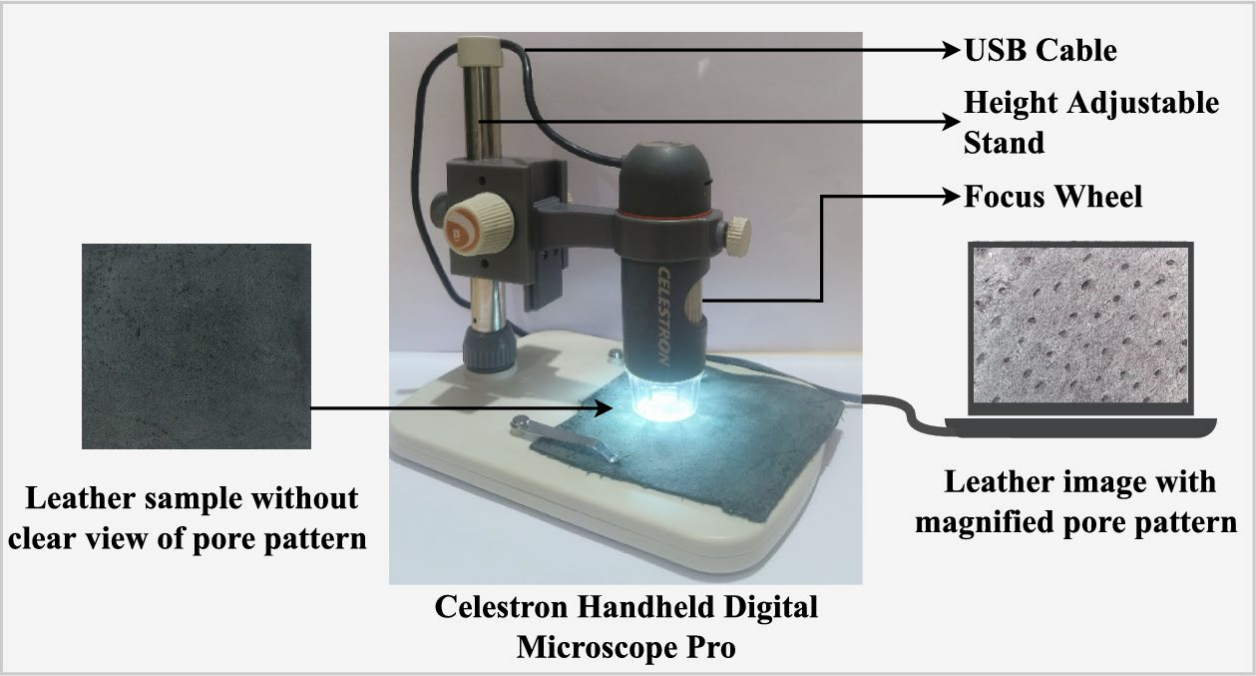
Pictorial representation of digital microscopic leather image acquisition^27^.

Selecting a digital microscope with adequate parameters is an essential step in image acquisition. In leather image analysis, the degree of magnification plays a vital role in the effective analysis of the leather grain surface. Concerning this, the leather samples are initially viewed under the Olympus SZ61 stereo-microscope. This experimentation helped identify the range of magnification power (10$\times$ to 150$\times$) over which the grain surface with well-magnified hair-pore patterns is viewed. Keeping this magnification range as a reference, to enable the visual experience of the species-unique grain surface, a high-resolution digital microscope with sufficient magnification is explored. Accordingly, the Celestron handheld digital Microscope Pro is chosen. It is a compact, cost-effective, and portable digital microscope with a 5MP sensor, an illuminator, a USB plug, a height-adjustable stand, and a focus wheel. Figure 2 shows the digital microscopic setup for leather image acquisition. The handheld microscope is connected to the computer using a USB cable. The software ‘Celestron MicroCapture Pro’ installed in the computer allows to view, capture, and save an image with .*jpg* extension. The height adjustable stand and the focus wheel of the microscope are adjusted to finely focus the sample at different magnifications ranging between 20$\times$ to 200$\times$. The software also has the option to set the sensor image resolution from the five resolutions: $1024 \times 1280$, $1080 \times 1920$, $1536 \times 2048$, $1744 \times 2320$, and $1944 \times 2592$. Accordingly, the pore patterns of a leather sample barely visible to the naked eye are clearly viewed using the Celestron handheld digital Microscope Pro. However, selecting an adequate degree of magnification and image resolution is an essential step.

#### Parameter selection

**Fig. 3:**
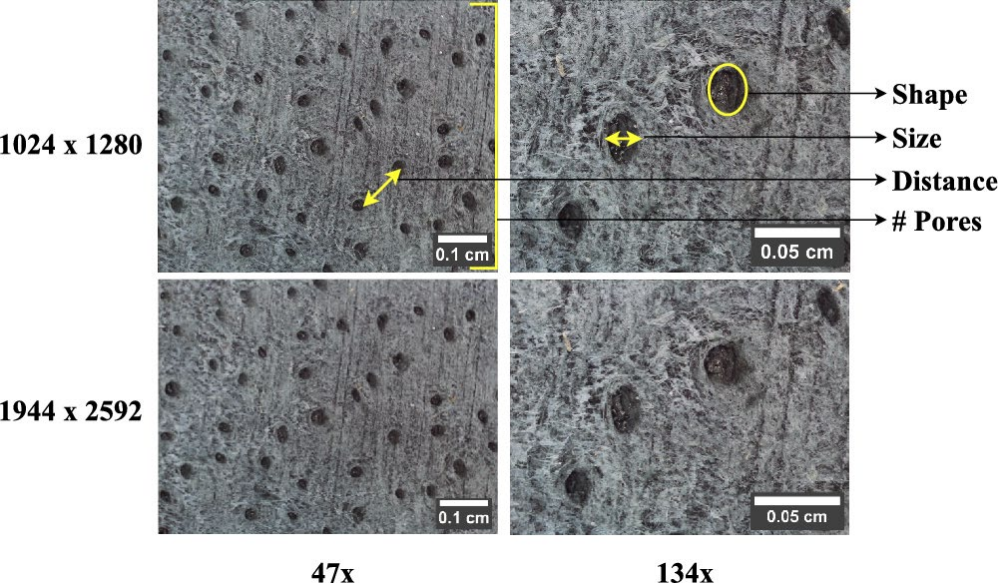
Leather images acquired at different magnification and image resolution (scale bar using ImageJ^28^).

An experiment is conducted to determine the image sensing parameters for leather image acquisition. A finished leather sample of buffalo hide with ideal behavior is used for experimentation. The microscope is directly placed on the sample, and the focus wheel is adjusted to get a fine focus. The pore patterns are definitely viewed at two focus points: $47\times$ and $134\times$. From Fig. 3, it can be noted that the leather sample viewed at $47\times$ magnification acquired significant number of hair-pores than the $134\times$ magnification. The existing study revealed that the number of hair pores, pore size and shape, and distance between the pores define the morphological behavior distinct to each species^7^. These features are jointly noticeable in $47\times$ images than $134\times$ images. Hence, $47\times$ is selected as an adequate degree of magnification for better analysis and estimation of morphological features. (ImageJ software is used to generate scale bars in the digital microscopic leather images^28^.)

The experiment also extends to determine the appropriate image resolution for a clear view of the grain surface. Figure 3 shows the leather images acquired at two resolutions: $1024 \times 1280$ and $1944 \times 2592$. Perceptually, the two images differ in the width and height pixel counts. Meanwhile, the image clarity is the same, which equals 4513 dpi, where dots per inch (dpi) is a quantitative metric that defines the picture clarity. However, the latter uses three times more memory than the former. Therefore, $1024 \times 1280$ image resolution is considerably convenient to clearly capture the hair-pore pixels. It also meets the memory requirements for further image analysis. With these parameter specifications, the microscope captures a rectangular area of $50~mm \times 70~mm$ of the leather sample. This field of view (FoV) at $47\times$ magnification is acquired by directly placing the microscope on the leather sample with a working distance (WD) of 45 *mm*. (WD is the distance between the sample and lens.) These microscopic measurements are calibrated with an accuracy of $\pm ~2~mm - 3~mm$. Table 3 lists the digital microscopic specifications for leather image acquisition. Thus, the experimental study effectively visualized the species-distinct leather grain surface by selecting adequate digital microscopic parameters.

**Table 3:** Specification list for digital microscopic leather image acquisition.

Specification	Measurement
Magnification	$47 \times$
Resolution	$1024 \times 1280$
Field of View (FoV)	$50~mm \times 70~mm$
Working Distance (WD)	45 *mm*
Measurement Accuracy	$\pm ~2~mm - 3~mm$

#### Image acquisition

The novel and unique leather image data acquisition process is executed and assisted by the concerned leather experts of the Central Leather Research Institute (CLRI), Chennai, India. CLRI is a Government body authorized to certify the quality of a leather sample. They conduct research and development on preserving hides and skins, improving leather quality, controlling pollution, detecting defects in leather, etc. Despite different research, the automatic identification of species from leather images is lacking in CLRI. Meanwhile, the outcome of this study can effectively help leather experts in automatic and quick species prediction with accurate decisions.

**Fig. 4:**
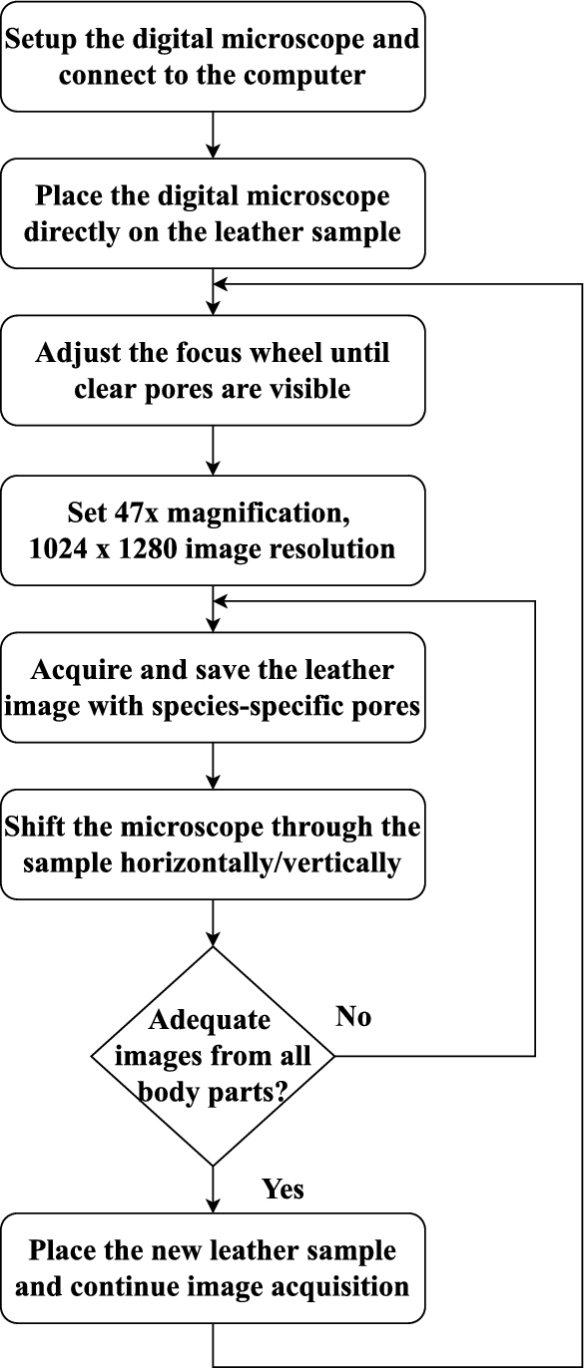
Flow diagram of the proposed digital microscopic leather image acquisition process.

Every day, the CLRI laboratory receives several leather samples to stamp the leather quality for global trade and product manufacture. The leather experts provided a few of these samples for leather image acquisition. The samples include the finished leather materials of buffalo and cowhides, goat and sheepskins. The buffalo and cowhides are larger, and their size is $\approx 85'' \times 86''$. Due to their larger size, they are mostly used in the manufacture of furniture. At the same time, the goat and sheepskins are smaller, and their size is $\approx 15'' \times 17''$. They are greatly used in smaller leather products such as shoe soles, belts, gloves, winter wear, etc. The cowhides are largely supplied among the four materials due to their exceptional strength and stretchability. Accordingly, the experts provided 20 buffalo hides, 51 cowhides, 32 goatskins, and 34 sheepskins, a total of 137 leather samples. Each animal skin is dyed/colored based on the final leather product requirement. Hence, to elevate diversity, different colored leather samples are acquired in this study. The acquisition process is initiated by setting up the microscope, placing it on the sample, and setting $47\times$ magnification and $1024 \times 1280$ image resolution. The acquisition setup and the chosen parameters have been approved by the leather experts. The images with varied pore characteristics are collected by shifting the scope all through the sample. Figure 4 pictorially describes the process of the leather image acquisition. Also, in a leather sample, the hair-pore patterns vary among different parts of the animal skin^6,29^. In other words, the body parts also influence the quality of the leather. Generally, skin includes six body parts: neck, shoulder, belly, butt, backbone, and shank, each with different hair-pore characteristics. Hence, this study also acquires digital microscopic leather images from six body parts. Thus, the novel and unique digital microscopic leather image data (LeaData) is created as a reference for species identification.

### Leather image behavior

The novel LeaData is a large pool of leather images with varied hair-pore patterns. It captures diverse leather images from different species, samples, and body parts. It, thereby, includes images with ideal and non-ideal behavior. The former comprises images with pore patterns that match the theoretical characteristics (literature). Meanwhile, the latter possesses leather images with practically challenging or distinctly varying pore pattern behavior.

#### Ideal behavior

**Fig. 5:**
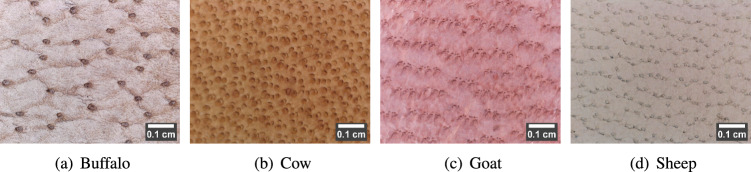
Ideal behavior of digital microscopic leather images per species (scale bar using ImageJ^28^).

Generally, in leather, the morphological behavior of hair pores characterizes each species^7^. The morphological behavior includes the size of the hair pore, the distance between the adjacent hair pores (inter-pore distance), the total number of pores per leather image, and the pattern of pore distribution. Figure 5 shows an example of species-specific novel leather images of different colors, and the following describes the ideal behavior of each species’ images:

i) The buffalo leather image possesses randomly scattered largest pores, as shown in Fig. 5a. It exhibits a sparsely distributed hair-pore pattern with the least number of pores per image and the highest inter-pore distance.
ii) The cow leather image is made of uniformly arranged medium-sized hair pores. The tight or densely packed hair-pore distribution with equidistant spacing between the pores is the unique and significant feature of the cow image. Due to this, it possesses the highest number of hair pores per image with the least inter-pore distance. Figure 5b portrays the uniformly spaced and densely packed hair-pore pattern in the cow image.
iii) The goat leather image is a mixture of medium and small-sized pores with a clustered arrangement. Each cluster contains closely packed three medium-sized pores, each with three small-sized pores, respectively. However, each cluster is widely spaced among the others. Hence, following the cow image, the goat image possesses comparatively lesser inter-pore distance with a moderate number of pores per image. Figure 5c depicts the clustered pattern of hair pores in the goat image.
iv) The sheep leather image comprises the smallest hair pores arranged in a wavery pattern, as shown in Figure 5d. Unlike the cow and goat images, the adjacent pores are less compactly spaced, exhibiting moderate inter-pore distance. Also, the number of pores per image is greater than the buffalo image and lesser than the goat image.

**Table 4:** Ideal characteristics of species-specific leather images.

		Buffalo	Cow	Goat	Sheep
1.	Pore size	Largest	Medium	Medium + Small	Small
2.	No. of pores	Least	Highest	Moderate	Less
3.	Pore pattern	Random	Dense	Cluster	Wavery
4.	Inter-pore distance	Highest	Least	Less	Moderate
5.	Vein-like structure	Highest	Least	High	Less

Therefore, ideally, the number of pores per image is highest in the cow, followed by goat, sheep, and buffalo images, respectively. Similarly, the pore size is the largest in the buffalo image, followed by goat, cow, and sheep. Table 4 presents the theoretical description (ideal behavior) of species-specific leather image behavior. The cow leather image carries substantially discriminating characteristics among the four species’ images. However, the sheep image behavior can often collide with the goat and buffalo images. Also, apart from pores, wrinkles or vein-like structures exist on the leather surface. As shown in Fig. 5, the veins are highly seen in the buffalo image, followed by goat, sheep, and cow, respectively. That is, as the number of pores increases, the presence of vein-like structures is less noticeable.

#### Non-ideal behavior

Practically, the species-specific pore characteristics exhibit inter-species similarity and intra-species variability. Such non-ideal behavior is due to the presence of non-specific pore sizes, varied pore patterns, overlapped pores, stretched/elongated pores, damaged pores, lightly visible pores, blurred and low contrast pores, etc. The following describes the practically challenging leather images with non-ideal behavior:

**Table 5:**
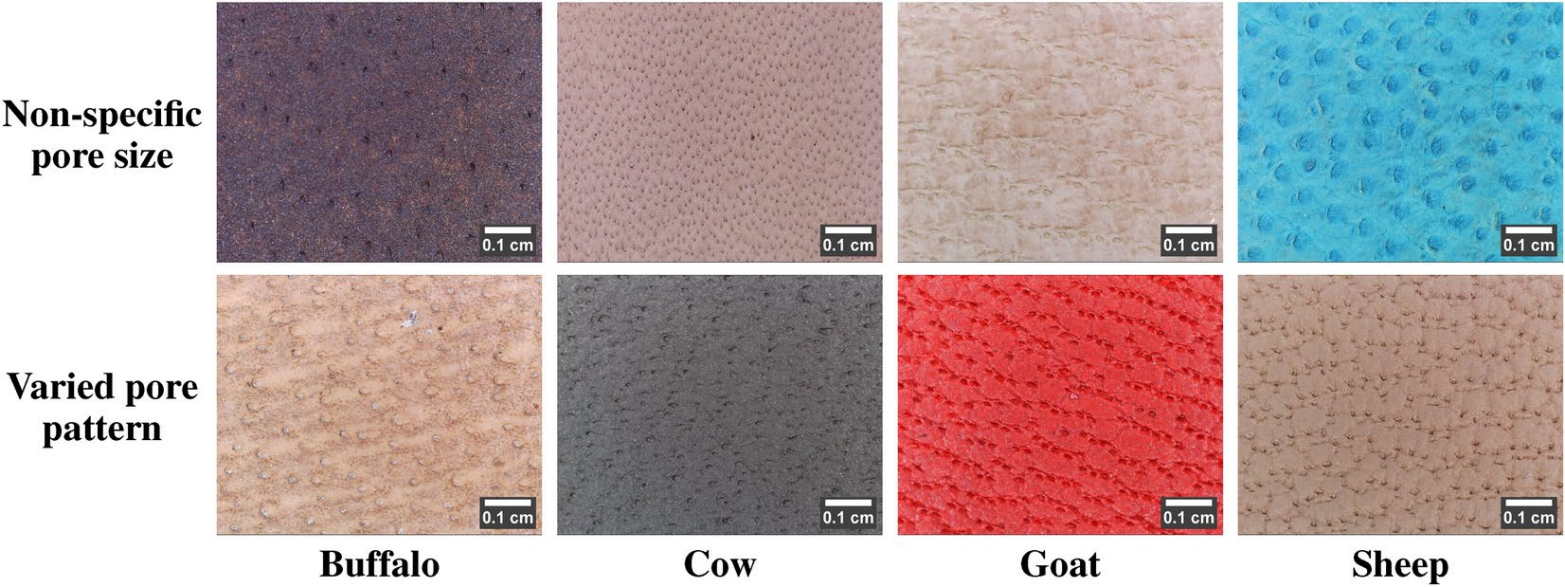
Leather images with non-ideal pore size and pore pattern (scale bar using ImageJ^28^).

**Table 6:**
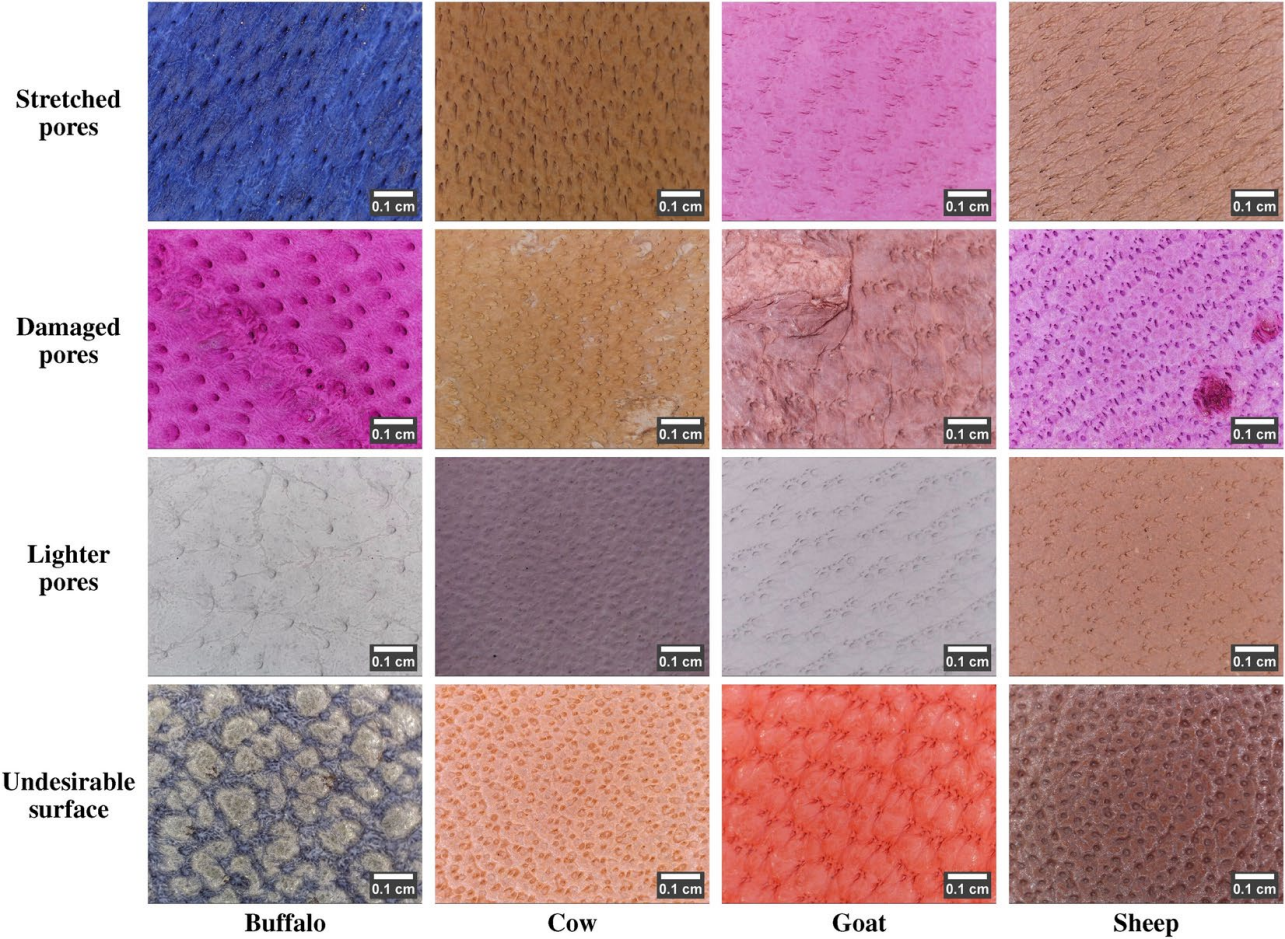
Non-ideal behavior of leather images due to leather-making process (scale bar using ImageJ^28^).

**Table 7:**
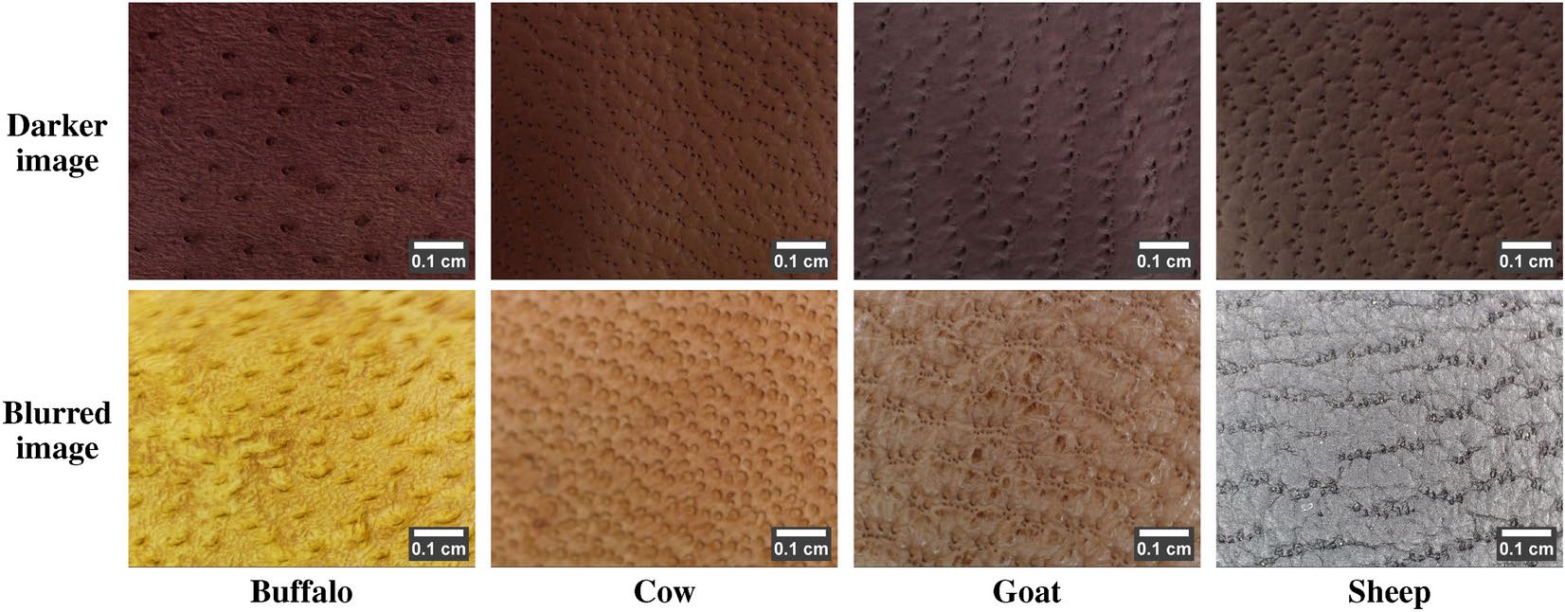
Non-ideal behavior of leather images due to leather image acquisition process (scale bar using ImageJ^28^).

i) The leather samples manufactured from animal skin belonging to different regions of locations, ages, breeds, etc., can carry different hair-pore sizes arranged in different patterns. Table 5 portrays an example of per-species leather images with vivid pore size and pattern. Ideally, the sheep leather image is said to have the smallest pores. However, in Table 5, row 1, the sheep image comprises the largest pores, similar to the ideal case in the buffalo image. At the same time, buffalo and cow images possess the smallest pores, and in the goat image, the medium-sized pores appear smaller than the ideal size. Meanwhile, in Table 5, row 2, the buffalo, cow, and goat leather images exhibit almost a wavery pattern similar to the sheep image. In contrast, the sheep image carries medium and small-sized pores, similar to the goat image, but in a wavery pattern.
ii) The leather-making process can often result in stretched/elongated pores, as shown in Table 6, row 1. The stretched pores are also more common in female animals. The leather-making process can also damage the leather surface, leading to damaged pores. In addition, animal birthmarks can give rise to damaged pores. In Table 6, row 2, the buffalo, cow, and goat images show the damaged pores due to the leather-making process. At the same time, the sheep image exhibits a portion of damaged pores due to an animal birthmark. Moreover, pores are tiny depressions and, hence, are assumed to be darker than the surface. However, due to the leather-making process, these depressions are buffed, making the pores lightly visible. Table 6, row 3 depicts the low-contrast leather images with lighter pores. In addition, during leather manufacture, the leather surface may also undergo undesirable changes, such as wrinkles, uneven dye distribution, puffed surface, etc. Table 6, row 4, portrays the hair pores distributed in the undesirable leather surface background.
iii) Practically, the pores of the leather samples dyed with darker colors (black, brown) are barely visible under high illumination. However, these samples acquired with bare illumination resulted in darker images with a relative contrast between the pore and surface. Table 7, row 1 depicts the darker leather images with darker pores. Also, during leather image acquisition, fewer images may also encounter image blur. Due to this, there exists a portion of the leather image with blurred pores, as shown in Table 7, row 2.

These non-ideal behaviors can misinterpret the morphological characteristics of a leather image. Using subjective analysis, they can also append the complexity of accurately determining the leather species. Therefore, the novel LeaData is created as a pool of leather images with ideal and non-ideal behavior. It can help in the objective analysis of leather image-specific characteristics for accurate, robust, and highly reliable decisions.

#### Body part-specific behavior

In a leather sample, the fiber weave and the hair-pore patterns differ from one part to the other. The consistent fiber weave results in a compact hair-pore pattern and vice-versa. The body part, starting from the neck edge to the tail with the thickest fiber weave that symmetrically divides the leather sample into two halves, is termed the ’backbone.’ The region halfway from the backbone and the belly with the consistent and rigid fiber weave is said to be the ’butt’. The sides of the butt with loosely bound fiber weave are the ’bellies’, and the region connecting the head is the ’neck’. Meanwhile, the region from the neck edge to one-third of the butt is the ’shoulder’, and the four sides of the animal skin above the knee to the shoulder/butt edge are the ’shank’.

**Fig. 6:**
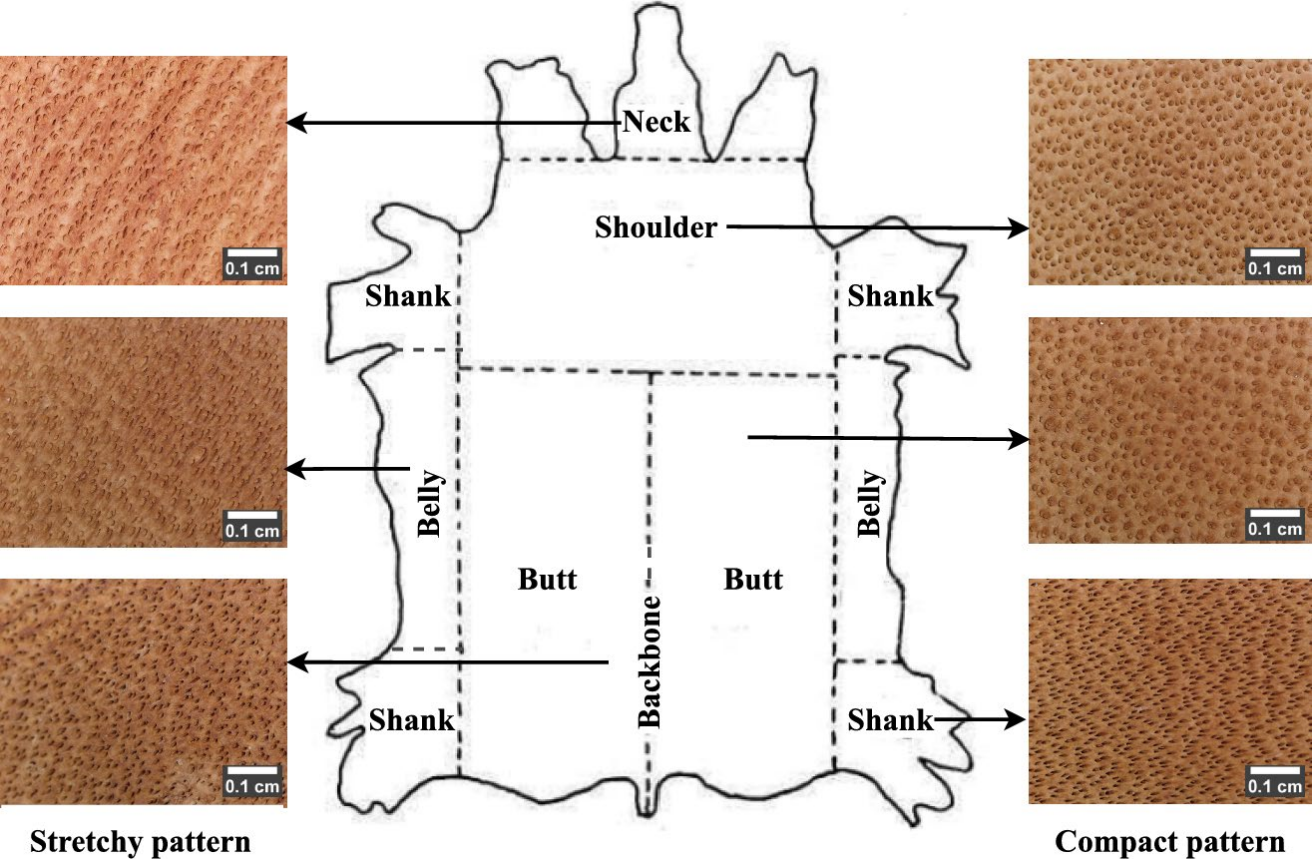
Example of body part-specific leather images (scale bar using ImageJ^28^).

On the whole, the fiber weaves are highly rigid in the butt, followed by the shoulder, shank, backbone, neck, and belly. Accordingly, the pores are more compactly arranged in the butt, while loosely bound and stretched pores are present in the belly. Figure 6 shows an example of a cowhide marked with different body parts. It illustrates examples of compact and stretchy pore patterns of cowhide. The degree of compact arrangement is the highest in the butt region, followed by the shoulder, and shank regions, respectively. Meanwhile, the stretchiness is lighter in the backbone, followed by the neck and belly, respectively. Therefore, due to the compact and rigid pore arrangement, it is more convenient to identify species from the butt region. However, it is practically challenging to determine species from the belly and neck regions. Therefore, the LeaData is created with body part-specific leather images to learn diverse pore behavior for reliable species prediction. Unlike the existing methods, it is a complete reference of leather images with varied characteristics.

## LeaData versions and analysis

In the field of digital image processing and computer vision, LeaData is a novel and unique digital microscopic leather image data. It is created for the automatic and quick identification of leather species by objectively analyzing the grain surface or hair-pore pattern. Table 8 lists the total number of digital microscopic leather images in the novel LeaData. It includes leather images from four species, 137 leather samples, and six body parts. Totally, it possesses 38,172 leather images acquired from the four permissible species: buffalo, cow, goat, and sheep. Among these, 4,256 are acquired from 20 buffalo hides, 22,684 from 51 cowhides, 6,101 from 32 goatskins, and 5,131 from 34 sheepskins, respectively. The data also span images from six body parts: backbone, belly, butt, neck, shank, and shoulder. Although the majority of the data, i.e., 19,925 leather images, are acquired from the butt region, and the remaining 18,247 images belong to other body parts. This is because, in general, leather experts examine the butt region with compact pore patterns to identify the species. However, the buffalo leather samples are unsymmetrically split into smaller pieces due to the larger size. Due to this, the backbone and shank regions are unavailable, and hence, as given in Table 8, their images are not collected in the LeaData. Despite this, the LeaData is an adequate reference for species-specific, body part-specific, and leather sample-specific leather images with ideal and non-ideal behavior. It is an umbrella of leather images with diverse behavior.

**Table 8:** Summary of species and body part-specific leather images in LeaData.

Leather Species	$\#$ Leather Samples	$\#$ Body Part-specific Leather Images						$\#$ Leather Images
		Backbone	Belly	Butt	Neck	Shank	Shoulder	
Buffalo	20	0	275	2659	707	0	615	4256
Cow	51	173	525	11333	3739	2836	4078	22684
Goat	32	422	478	3118	554	525	1004	6101
Sheep	34	313	301	2815	485	499	718	5131
	137	908	1579	19925	5485	3860	6415	**38172**

The novel LeaData acquisition process is compared and analyzed with the existing processes. Table 9 compares the SEM^7^, digital camera^20^, and proposed digital microscopic leather image analysis. The SEM is a heavily bulky and highly expensive setup. The data curation/acquisition process is complicated and expert-dependent. At the same time, the digital camera-captured leather image analysis using an RTI setup is comparatively less complex than the SEM. Also, the acquisition process is lightly complex. However, the selection of desirable magnification is lacking. Meanwhile, the proposed setup is the simplest and can be easily handled without expert dependency. It is the cheapest among the SEM and RTI + camera-based imaging devices and is capable of capturing well-magnified images. The SEM-based image is highly magnified, with a few/insufficient pores and patterns on the leather surface. The digital camera-based leather images possess pore patterns with unclear pore boundaries. Nevertheless, the digital microscopic leather images capture a comparatively greater number of pores with clear patterns and boundaries. Moreover, due to the simple acquisition process, the proposed method created adequately bigger leather image data for accurate and reliable species interpretation. Therefore, unlike the traditional leather image analyses, the present LeaData establishes an extensive and exhaustive reference study of hair-pore patterns for automatic species identification.

**Table 9:** Comparative analysis of proposed and existing leather image analyses.

	SEM-based leather image analysis^7^	Digital camera-based leather image analysis^20^	Digital microscopic leather image analysis (proposed)
Imaging Device	Scanning electron microscope (SEM)	RTI + digital camera	Digital microscope
Acquired Leather Image	Insufficient pore regions with indistinct pore patterns	Sufficient pores with indistinct pattern and unclear pore boundaries	Sufficient pores with distinct pattern and clear pore boundaries
Setup	Heavily bulky (laboratory-specific)	Lightly bulky (laboratory-specific)	Simpler (portable and handheld)
Cost- Effectiveness	Highly expensive (cost-ineffective)	Expensive (cost-ineffective)	Cheaper (cost-effective)
Magnification	Highly magnified (100 $\times$)	Uncertain magnification	Well-magnified (47 $\times$)
Image Acquisition Process	Comparatively complex and tedious	Lightly complex and tedious	Simple and easy
Data Adequacy	Inadequate	Inadequate	Adequate (big data)

### LeaData organization and versions

**Fig. 7:**
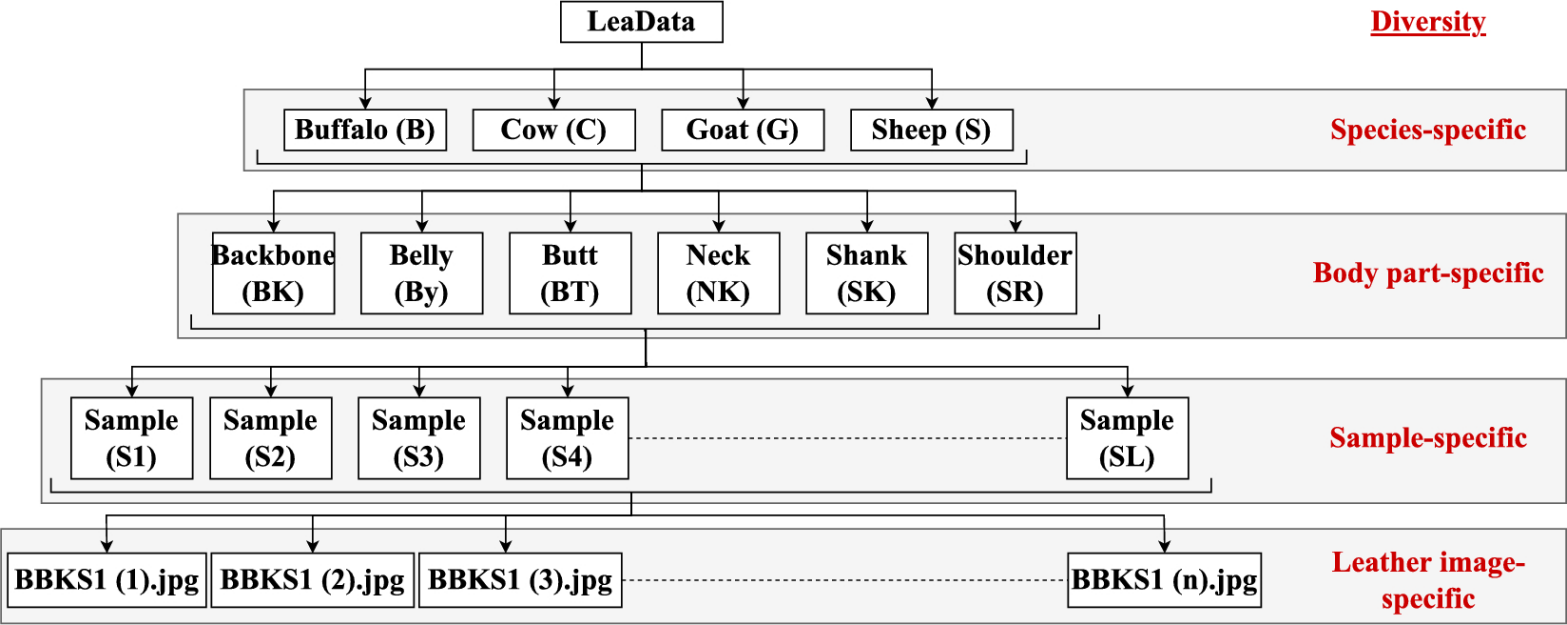
Schematic representation of LeaData organization.

In LeaData, the 38,172 leather images are organized into four classes (species): Buffalo, Cow, Goat, and Sheep. Figure 7 pictorially illustrates the LeaData organization. The filename of an image belonging to the Buffalo class begins with *B*, while *C* for Cow, *G* for Goat, and *S* for Sheep, respectively. Further, each class is subdivided into six sub-classes (body parts): Backbone, Belly, Butt, Neck, Shank, and Shoulder. The filename of an image belonging to the Backbone of the Buffalo class appends *BK* with *B*, i.e., *BBK*, while *BBY* for Buffalo Belly, *BBT* for Buffalo Butt, *BNK* for Buffalo Neck, *BSK* for Buffalo Shank, and *BSR* for Buffalo Shoulder, respectively. Moreover, each sub-class is grouped into *L* leather samples ranging from *S*1 to *SL*. Hundreds of images are captured from each sample to obtain pore variations with different orientations and non-ideal behavior. The $n^{th}$ image belonging to the leather sample 1 of the Backbone of the Buffalo class has the following filename: *BBKS*1 (*n*).*jpg*. (where .*jpg* is the format in which the Celestron handheld digital microscope saves an image.)

The LeaData mostly include the cow images (22,684) captured from the larger number of cowhides (51 samples). Also, due to the larger sample size, the buffalo and cowhides are divided into two halves, and the images are acquired from one half of the sample. Therefore, fewer images are acquired from the backbone, belly, and shank regions of buffalo and cowhides. Moreover, the butt region is generally examined for species identification, and hence, the images are mostly from the butt body part. Hence, the LeaData is further split into three LeaData versions: LeaData-V1, LeaData-V2, and LeaData-V3. The three versions are built with leather images exhibiting ideal, non-ideal, and body part-specific behavior, respectively.

#### LeaData-V1

LeaData-V1 is a subset of LeaData with leather images exhibiting ideal pore characteristics. It is small-scale data with only 1,200 leather images equally divided into four species. In other words, it is balanced data with each species carrying 300 images, respectively. These ideal images are chosen from the butt region of five buffalo hides, two cowhides, three goatskins, and six sheepskins, respectively. It also includes a 10% of practically challenging leather images. They carry certain non-ideal characteristics, such as the leather surface with wrinkles and damaged and slightly elongated pores. Therefore, LeaData-V1 is the most convenient for analyzing the ideal morphological characteristics of each species’ image. It is well-suited to apply digital image processing (DIP) and ML techniques for smaller data analysis. However, it is unsuitable for analyzing leather images with non-ideal characteristics.

#### LeaData-V2

The LeaData-V2 is another subset of LeaData with a comparatively larger number of leather images. It is a balanced data with 7,600 images, created as a reference for non-ideal leather image behavior. Each species contains 1,900 images acquired from eight buffalo hides, seven cowhides, fifteen goatskins, and sixteen sheepskins, respectively. In other words, it encloses diverse leather image behavior from 46 vivid leather samples. Therefore, comparatively, LeaData-V2 is $\approx$ 2.8 times more diverse than LeaData-V1. It is best suited for sample-specific leather image analysis.

**Fig. 8:**
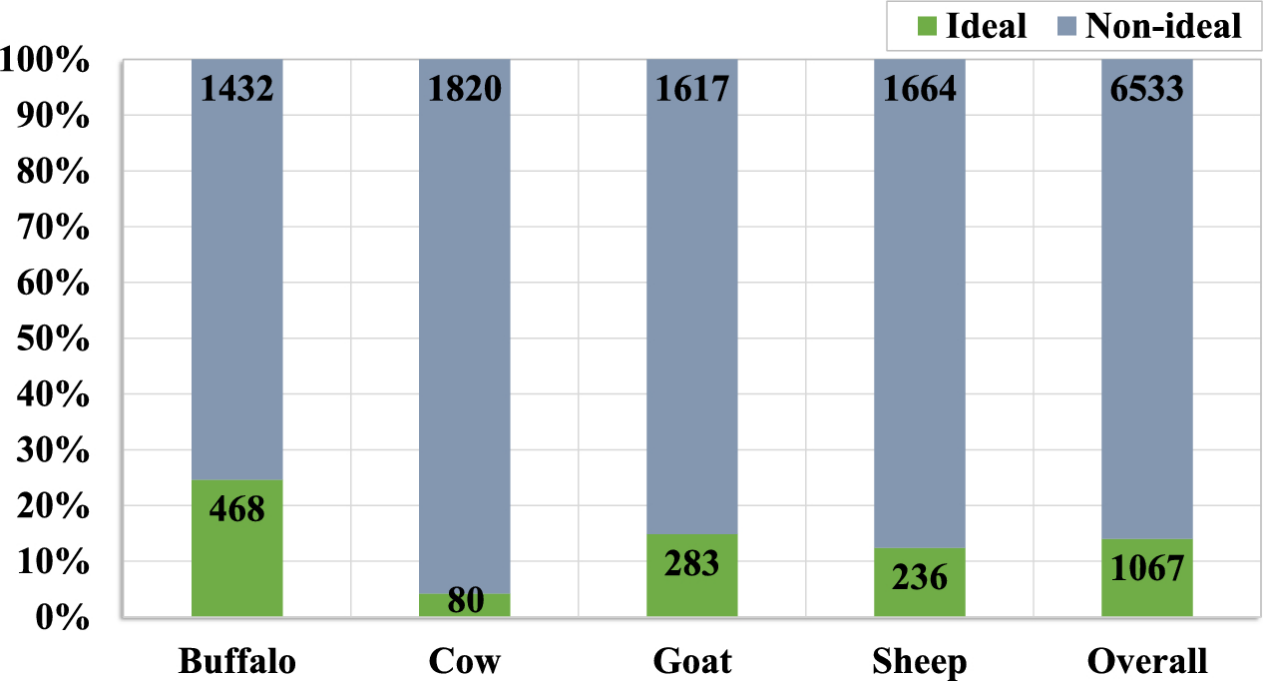
Per species ideal and non-ideal leather images in LeaData-V2.

In LeaData-V2, the entire 7,600 leather images are chosen from the butt region. Yet, most images do not carry the ideal compact pore pattern, instead with non-specific pore size and varied pore pattern behavior. It also comprises leather images of the butt region with stretched and damaged pores as an effect of the leather-making process. Moreover, it holds low-contrast and darker leather images with lighter, darker, and burred pores. Therefore, LeaData-V2 includes 6,533 leather images (86%) with practically challenging, complex, and non-ideal behavior. The remaining 1,067 images (14%) exhibit ideal characteristics. Figure 8 illustrates the percentage of per-species leather images with ideal and non-ideal behavior in LeaData-V2. Among 1,900 buffalo leather images, 468 (25%) are ideal, and 1,432 (75%) are non-ideal. Meanwhile, only 80 (4%) cow leather images are ideal, and the rest, 1,820 (96%), are non-ideal. At the same time, 283 (15%) goat and 236 (12%) sheep leather images are ideal, and the remaining 1,617 (85%) and 1,664 (88%) of them are non-ideal, respectively. Therefore, LeaData-V2 is a source of practically challenging leather images with inter-species similar and intra-species variable characteristics. It is a reference for species and sample-specific leather images with non-ideal (diverse) behavior. Exploring various DL techniques for larger data analysis using LeaData-V2 is also convenient.

#### LeaData-V3

Unlike LeaData-V1 and LeaData-V2, the LeaData-V3, a subset of LeaData, comprises species-specific leather images from different body parts. It mimics the LeaData but with a balanced number of images in each species. A total of 10,000 leather images grouped into four species are enclosed in LeaData-V3. The images are chosen from 16 buffalo hides, 13 cow hides, and 20 goat and sheepskins, respectively. In other words, LeaData-V3 holds diverse leather images from 69 different leather samples, which is $\approx$ 4.3 and 1.5 times more diverse than LeaData-V1 and V2, respectively.

**Fig. 9:**
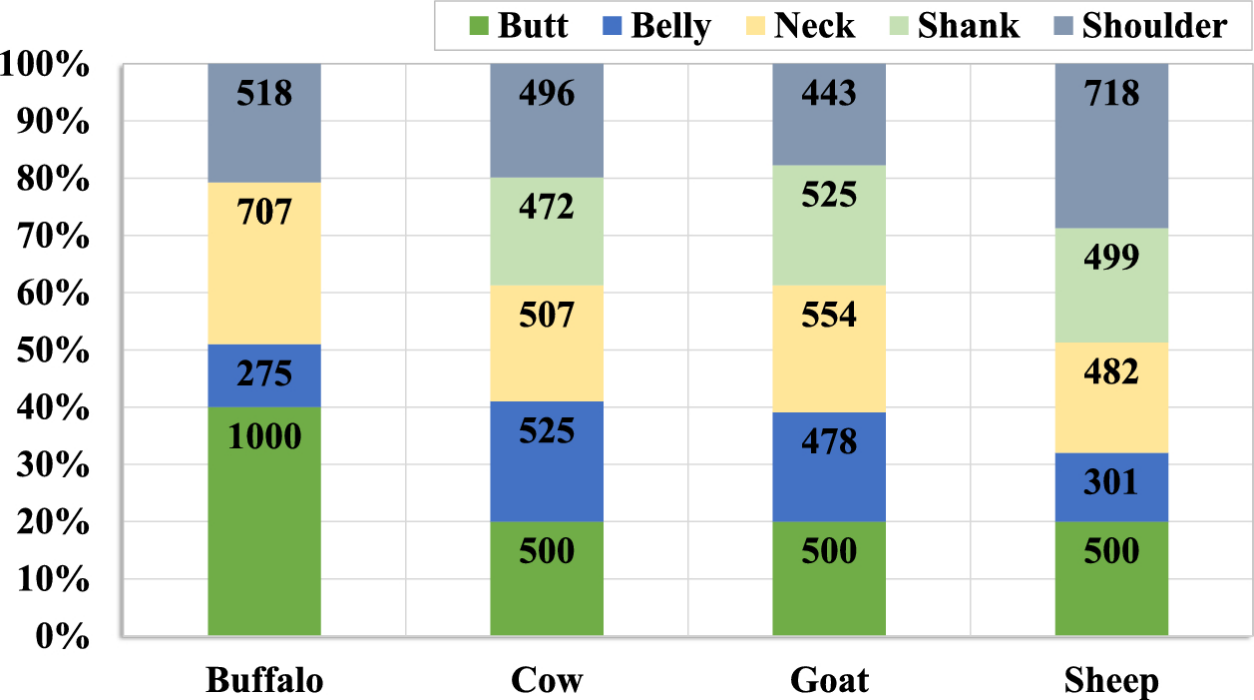
Per species body part-specific leather images in LeaData-V3.

Moreover, in the LeaData, the backbone images are the least, and their number is greatly unbalanced between the species. Learning the species-specific pore characteristics from the lesser number of backbone images is insufficient, and hence, they are not included in the LeaData-V3. That is, the LeaData-V3 spans leather images from five body parts: butt, belly, neck, shank, and shoulder. It comprises 2,500 leather images in each of the four species unequally distributed into five body parts. Figure 9 presents the percentage of per species leather images distributed over each body part. Totally, there are 2,500 butt images, 1,579 belly images, 2,250 neck images, 1,496 shank images, and 2,175 shoulder images. The butt leather images are selected from the leather samples different from that of the LeaData-V1 and LeaData-V2 sets. The buffalo species contains 1,000 images from the butt and 1,500 from other body parts. However, it does not possess shank images due to unsymmetrical sample division. At the same time, 472, 525, and 499 shank images are acquired from the cow, goat, and sheep leather samples, respectively. Similarly, very few belly images are found in the buffalo species, followed by sheep, goat, and cow. Meanwhile, more neck and shoulder images are seen in the buffalo and sheep species, respectively. The rest of the species possesses a comparable number of neck and shoulder images. Therefore, LeaData-V3 is a reference for species, body-part, and sample-specific leather image behavior with balanced data. It is also suitable for DL techniques with larger data analysis.

Therefore, in leather technology, LeaData and its versions are digitized forms of permissible leather materials. They help analyze vivid grain pattern characteristics, ideal and non-ideal, objectively without expert dependency. Moreover, unlike conventional methodologies, the LeaData analysis can reduce false leather species interpretations. Thus, LeaData can form a strong base or reference to establish a smart leather species identification technique.

### LeaData analysis

Generally, digital microscopic images carry information regarding their pattern and texture. In LeaData, the digital microscopic leather images convey species-unique hair pore patterns distributed over the leather surface texture. Digital image processing is an efficient way to automatically process, manipulate, and mathematically interpret the visual information contained in the images. At the same time, an automatic learning algorithm, ML/DL, is a powerful tool for optimizing and automating the classification of data into respective classes. Hence, recently, a few studies have demonstrated LeaData analysis by intertwining DIP with ML/DL for the extraction, quantification, and classification of the species-definite leather image features into permissible species. Table 10 summarizes the methodologies and results of the recent studies conducted on novel and unique leather images.

**Table 10:** Summary of recent methodologies using the novel LeaData analysis.

Method	Pore feature analysis			Texture analysis	Transfer learning		Full-training
Pre-processing	Scale + shift	CS+AHE, MAU	–	–	–	–	–
Segmentation	Otsu’s	EOCA	ApSnet	–	–	–	–
Feature Extraction	CHT (M)	CCA (MGS)	CCA (MGS)	Texture (DWT+LBP)	Rich Features (ResNet18)	–	–
Classification	KNN	MLP	MLP	MLP	MLP	Fine-tuning (ResNet18)	Inception V3
Accuracy on LeaData-V1	92.5$\%$	98.75$\%$	99.58$\%$	99.58$\%$	99.58$\%$	99.69$\%$	98.5$\%$
LeaData-V2	–	–	-	–	–	–	98.5$\%$
Complexity	Complex	Very Complex	Complex	Moderate	Simple	Moderate	Complex
Generalization Ability	Least	Least	Less	Lesser	Moderate	High	Higher

Initially, different DIP techniques such as pre-processing, segmentation, feature extraction, and ML algorithms are applied to LeaData-V1 for ideal leather image analysis. As the color in the leather images is not a species-distinguishing feature, the analyses are initialized by converting the images to gray-scale. A simple scaling and shifting-based image pre-processing, Otsu’s thresholding-based image segmentation, followed by circular Hough transform (CHT), quantified the morphological behavior of leather images^24^. The extracted morphological image features: pore radius, inter-pore distance, and number of pores per image were on par with the theoretical fact. However, the k-nearest neighbor (KNN) based feature classification gave 92.5% accurate species prediction. On the other hand, the hybrid and complex DIP technique with multi-layer perceptron (MLP) based feature classification improved the prediction rate to 98.75% accuracy^30^. This improvement is due to the effective feature extraction facilitated by efficient pre-processing and segmentation approaches. That is, the contrast stretching plus adaptive histogram equalization (CS + AHE) based image enhancement and the median, averaging, and unsharp masking (MAU) based image filtering effectively highlighted the useful pore regions. Followed by, the Entropy-based Otsu’s thresholding with Component-area-histogram Analysis (EOCA) rightly isolated the species-specific hair-pores. Moreover, the extraction of seventeen image features using connected component analysis (CCA) quantified the morphological, geometrical, and statistical (MGS) characteristics of the leather images. The combined feature analysis elevated the species prediction. However, the analysis is greatly complex and confined to a specific set of images. Meanwhile, the auto-pore segmentation network (ApSnet) followed by MLP-based classification offered automatic species identification with better generalization^27^. Due to the DL-based adaptive and automatic learning ability, the pore regions were better isolated without the hybrid pre-processing step. It, thereby, substantially improved the feature extraction followed by classification with 99.58% accuracy. Thus, the DIP and ML-based feature analysis objectively determined and validated the ideal pore characteristics for accurate species prediction. However, it is complicated and restricted to pore feature analysis, and hence, it is challenging to determine species from leather images with corrupted/damaged pores.

Texture analysis is an efficient way to jointly analyze the foreground pore and background leather surface characteristics^31^. This property provides better generalization than pore feature analyses. Moreover, it is less complex as it is independent of pre-processing and hair-pore segmentation. Among different texture analysis methods, discrete wavelet transform (DWT) based structural approach and local binary pattern (LBP) based statistical approach are the most common. The MLP-based leather image texture analysis gave 96.25% with the DWT features and 97.08% with the LBP features. However, their combination improved the accuracy to 99.58%, which is on par with the pore feature analysis. That is, the texture analysis determined the efficiency of pore and leather surface for accurate species prediction. However, the texture features are hand-crafted for a specific task and, hence, lack in generalization. On the other hand, DL can adaptively learn the leather image-specific characteristics and offer better generalization. Hence, the convolutional neural network (CNN) based DL approach is employed to quantify the leather image features called rich features^32^. Transfer learning is applied to the pre-trained ResNet18 for rich feature extraction. Followed by, the support vector machine (SVM) based rich feature classification offered 99.58% accurate prediction. The results are on par with the texture and pore feature analyses at the cost of high-dimensional data. Also, the identification technique is semi-automated due to separate feature extraction and classification processes. In contrast, transfer learning-based fine-tuning of ResNet18 resulted in a fully automated leather image classification system integrated with feature extraction and classification^33^. It resulted in 99.69% accurate species prediction with better generalization. Yet, the technique is restricted to ideal leather image analysis learned from smaller LeaData-V1.

Full-training CNN with larger LeaData-V2 can improve the learning ability to ideal and non-ideal characteristics^34^. Among different CNNs, the complex InceptionV3 accurately predicted 98.5% of leather images with ideal and non-ideal behavior. The technique with diverse leather image analysis offered robust and highly reliable results at the cost of computationally complex network architecture and a tedious learning process. Therefore, unlike the existing methods, the recent methodologies with the novel LeaData developed an automatic leather species identification technique with accurate results. It effectively predicted species from leather images with ideal and non-ideal characteristics. Also, comparatively, it is easier, faster, and less prone to false predictions. Nevertheless, learning from LeaData-V3 with body part-specific leather image behavior can further offer more robust results. Also, expanding the LeaData with the leather images from different region-of-locations can further elevate the knowledge of diverse leather behavior.

## Conclusion

In leather, examining the grain surface with hair-pore distribution is a simple way to identify species. Hence, this work proposed the digital microscopic leather image analysis. It solely created and analyzed the novel and unique leather image data (LeaData) with well-magnified species-unique grain patterns. A simple and compact commercially available Celestron handheld digital microscope pro with 47$\times$ magnification is equipped for leather image acquisition. The grain surface images of the buffalo, cow, goat, and sheep leather samples with ideal and non-ideal characteristics are acquired. Moreover, diversity is provided by including images from 137 leather samples and six body parts. The three versions of LeaData: LeaData-V1 with 1,200 images, LeaData-V2 with 7,600 images, and LeaData-V3 with 10,000 images, provided a complete reference study on the leather images with ideal, non-ideal, and body part-specific behavior. The leather images are a novel contribution to the fields of DIP and computer vision to establish digitization in leather technology. Research on leather image analysis can assist leather specialists, product manufacturers, and customs officials in easy and quick species determination. In the future, it may be of great interest worldwide. Hence, the LeaData-V1 and LeaData-V2 are made openly accessible in the Dryad data repository. The researchers can download them for research purposes via the following DOI: https://datadryad.org/stash/share/kZMqZ-q817jk4v-cKsJs7XvPzeBo-c0Pw_a90-VaYqs. Meanwhile, the LeaData-V3 will be provided upon request. Therefore, this article is a complete database paper on the novel LeaData with the following contributions:

i) Detailed review of the existing/traditional leather species identification techniques and their challenges.
ii) Simple, easy, and quick way of leather image acquisition process using a digital microscope.
iii) Diverse LeaData description based on the organization, versions, and behavior.
iv) Open access of novel LeaData and its versions in the Dryad data repository, making it freely discoverable and citable for research purposes.
v) Research promotions on digital microscopic leather image analysis using LeaData by highlighting the recent studies and the scope of improvement.

### Future scope

Therefore, the novel LeaData is a digital platform for researchers in leather technology for automatic species identification. They can not only use this data to determine the animal species in leather but also to certify its authenticity. In other words, LeaData can also assist the leather specialists and DIP specialists in automatically categorizing the leather products manufactured from actual leather or non-leather. Furthermore, it can also be used as a basis for the automatic identification of a leather image with defects. That is, researchers can also employ the LeaData to detect defects automatically in leather. Moreover, the LeaData encloses leather images of permissible materials restricted to India. However, the permissibility of leather may vary from one country to another. Therefore, researchers can employ LeaData to assist the customs officials in automatically determining Indian leather goods. They can further expand the dataset with the leather images acquired from different countries to differentiate between the country-based leather materials. Therefore, the scope of LeaData is not restricted to species identification but also to leather authenticity, defect detection, etc. Its outcome has a great impact on biodiversity preservation that helps in manufacturing and trading the leather from permissible material while protecting the exotic animal skin. It also ensures consumer protection by trading leather products from high-quality, defect-less, authentic leather materials.

## Data Availability

Yes, the data is openly accessible in the Dryad data repository, and it can be downloaded using the following link: https://datadryad.org/stash/share/kZMqZ-q817jk4v-cKsJs7XvPzeBo-c0Pw_a90-VaYqs. However, the data is confidential, and hence, it can be used with an agreement not to use it for any commercial purpose.
